# Editorial: Vibrio Virulence Regulation and Host Interactions

**DOI:** 10.3389/fcimb.2021.793464

**Published:** 2021-10-29

**Authors:** Lixing Huang, Yang Fu, Wenxiang Sun, Joanna Brzostek

**Affiliations:** ^1^ Fisheries College, Fujian Engineering Research Center of Aquatic Breeding and Healthy Aquaculture, Jimei University, Xiamen, China; ^2^ School of Medicine, Southern University of Science and Technology, Shenzhen, China; ^3^ Huntsman Cancer Institute and Department of Pathology, School of Medicine, University of Utah, Salt Lake City, UT, United States; ^4^ Department of Microbiology and Immunology, National University of Singapore, Singapore, Singapore

**Keywords:** vibrio, virulence, pathogen-host interactions, Research Topic, bacteria

*Vibrio* is an ubiquitous bacterium that is widely present in various aquatic and marine habitats; among the more than 100 species of *Vibrio* described, about 12 can cause human infections, while other *Vibrio* lead to marine animal infections. *Vibrio cholerae* can cause cholera, a serious diarrheal disease that is life-threatening if left untreated, and usually spread through contaminated water and personal contact. Non-cholera *Vibrio* species cause vibriosis - an infection usually are acquired through contacting with seawater or eating raw or undercooked contaminated seafood. Non-cholera *Vibrio* can cause a variety of clinical manifestations, including self-limiting gastroenteritis, wound infection, sepsis, and death. The incidence of vibriosis is on the rise, partly due to the spread of *Vibrio* spp. favored by climate change and rising sea temperature.

Considering that *Vibrio* is the leading opportunistic pathogen causing serious infections in humans and marine animals, there is an urgent need to better understand the pathogenic mechanism of *Vibrio* infections to guide effective prevention and treatments. Meanwhile, preventing and avoiding *Vibrio* infections and the resulting deaths require a deeper understanding of the mechanisms by which human and animal immune systems recognize and respond to *Vibrio*. To this end and through this Research Topic of 12 articles including original research and comprehensive reviews, we coalesce the latest advances in the current understanding of *Vibrio* virulence regulation and host interactions.

## *Vibrio cholerae* Virulence Regulation and Host Interactions

*V. cholerae* is the pathogen causing cholera. It can proliferate in the water environment and infect humans through contaminated food and water. In *V. cholerae*, virulence is a multilocus phenomenon with a large functionally associated network. Ramamurthy et al. extensively summarize the regulation of important virulence factors in *V. cholerae* and the host response in the context of pathogenesis, covering topics such as major toxins produced by *V. cholerae* and their regulation, the control of virulence by the ToxR regulon, the regulation of secretory systems, the host’s response to toxins and somatic antigens, as well as the host’s inflammatory response. In addition, Kumar et al. evaluate and discuss our current understanding of the different functions of *Vibrio* pathogenicity island-1. In *V. cholerae*, this is related to virulence, important for toxin production, and essential for disease development. Biswas et al. summarize the diguanylate cyclases (DGCs) identified in *V. cholerae* so far, and emphasize the importance of DGCs and their product c-di-GMP in the virulence and lifecycle of *V. cholerae*.

Increased attention is being paid to the fact that the large number of microorganisms residing in human gastrointestinal tract establish a special micro-ecological system, which immediately responds to the invasion of *V. cholera* through the mechanism of “colonization resistance”, such as the production of antimicrobial peptides, the competition of nutrients, and maintenance of the intestinal barrier. At the same time, *V. cholerae* can quickly sense these signals and regulate the expression of related genes to avoid stresses during infection, thereby successfully colonizing the surface of small intestinal epithelial cells. The comprehensive review by Qin et al. summarizes the interactions between the gut microbiota and *V. cholerae* in terms of type VI secretion system, quorum sensing, reactive oxygen species/pH stress, and bioactive metabolites.

## *Vibrio parahaemolyticus* Virulence Regulation and Host Interactions

*V. parahaemolyticus* is a common pathogenic marine bacteria that can cause gastrointestinal infections and other health complications, posing a life threat to patients with weakened immune functions. In the past two decades, the pathogenicity of environmental *V. parahaemolyticus* has greatly increased. With more frequent studies showing the fast-evolving nature of *V. parahaemolyticus*, in-depth evaluation of its pathogenic ability becomes imperative. In this regard, Liu et al. demonstrate that the difference in pathogenicity of environmental *V. parahaemolyticus* strains is caused by a combination of HGT level, pathogenic elements distribution, and secretory system properties. Therefore, it is necessary to further study the genomic differences between environmental and clinical *V. parahaemolyticus* in order to better understand the pathogenicity of *V. parahaemolyticus*.

An in-depth understanding of the non-classical virulence genes of *V. parahaemolyticus* will help to better understand its pathogenic mechanism. Some manuscripts in this Research Topic reflect this view. For example, Pérez-Reytor et al. identify a gene encoding zonula occludens toxin (Zot) in the genome of highly cytotoxic strains of Chilean *V. parahaemolyticus*, which contributes to the virulence of *V. parahaemolyticus* through disturbance of the actin cytoskeleton. Yang et al. investigate the function of a prophage-related gene named VpaChn25_0724 in *V. parahaemolyticus* CHN25. Their results reveal that VpaChn25_0724 contributes to the cell membrane integrity and growth of *V. parahaemolyticus*.

## Virulence Regulation in Other *Vibrios*

*V. harveyi*, *V. alginolyticus* and *V. vulnificus* are the chief cause of vibriosis and have led to high mortality of marine animals and severe global economic losses. Understanding the virulence regulatory mechanisms of these *Vibrios* is the key to better development of fish vaccines. To this end, Xu et al. show that chemotactic genes *cheA*, *cheB*, *cheR*, *cheV* and *cheY* can regulate the adhesion ability of *V. harveyi* by affecting motility, and participate in the adjustment of adhesion at different temperatures, salinities and pH. Zeng et al. analyze the succinylome of *V. alginolyticus* for the first time and reveal the possible biological effects of lysine succinylated protein, of which 7.5% is predicted to be a virulence factor so it may provide possible targets for the development of attenuated vaccines. Gong et al. reveal that *V. vulnificus* RpoS regulates TolCV1 expression, thereby affecting the secretion of RtxA1, bile salt tolerance and lethality in mice.

## Virulence and Gene Diversity of *Vibrio* spp

*Vibrios* are an abundant and diverse group of bacteria in marine and estuarine environments, and there is evidence of biochemical and genotypic correlations with virulence potential. In this regard, Lydon et al. determine the biochemical characteristics and virulence genotypes of 30 clinical and 39 oyster isolates of *V. vulnificus* based on the types of 16S rRNA gene (*rrn*) and virulence-related gene (*vcg*), which identify a relationship between isolates from the cooler season and systemic virulence potential in *V. vulnificus*. Besides, Wu et al. determine the genomic sequence of T3SS effector-encoding regions from 62 strains of four different species, and clarified the variation of their effector repertoires. In addition, they found a potential novel effector in *V. harveyi* and *V. campbellii* strain, and revealed that differences in T3SS-mediated cytotoxicity not only dependent on variation in the *Vibrio* T3SS effector repertoires, but also initial adhesion ability to host cells.

## Concluding Remarks

The primary articles and reviews featured in this Research Topic are great examples presenting the current state of knowledge in this exciting and fast developing field of *Vibrio* spp. virulence regulation and host interactions. Through each new article, we gain a deeper understanding of the interesting and unique mechanisms controlling *Vibrio* pathogenicity and the pathogen-host interactions. In turn, these studies will contribute to the formulation of vibriosis control strategies and the development of highly effective live attenuated vaccines.

## Author Contributions

All authors listed have made a substantial, direct and intellectual contribution to the work, and approved it for publication.

## Funding

This work was supported by grants from the Natural Science Foundation of Fujian Province under contract No. 2019J06020 and the National Natural Science Foundation of China under contract No. 32173016.

## Conflict of Interest

The authors declare that the research was conducted in the absence of any commercial or financial relationships that could be construed as a potential conflict of interest.

## Publisher’s Note

All claims expressed in this article are solely those of the authors and do not necessarily represent those of their affiliated organizations, or those of the publisher, the editors and the reviewers. Any product that may be evaluated in this article, or claim that may be made by its manufacturer, is not guaranteed or endorsed by the publisher.

